# Prognostic Value of Choline and Other Metabolites Measured Using ^1^H-Magnetic Resonance Spectroscopy in Gliomas: A Meta-Analysis and Systemic Review

**DOI:** 10.3390/metabo12121219

**Published:** 2022-12-05

**Authors:** Yixin Shi, Delin Liu, Ziren Kong, Qianshu Liu, Hao Xing, Yuekun Wang, Yu Wang, Wenbin Ma

**Affiliations:** 1 Department of Neurosurgery, Center for Malignant Brain Tumors, National Glioma MDT Alliance, Peking Union Medical College Hospital, Chinese Academy of Medical Sciences and Peking Union Medical College, Beijing 100730, China; 2 Eight-Year Medical Doctor Program, Chinese Academy of Medical Sciences and Peking Union Medical College, Beijing 100730, China; 3 Department of Head and Neck Surgery, National Cancer Center/National Clinical Research Center for Cancer/Cancer Hospital, Chinese Academy of Medical Sciences and Peking Union Medical College, Beijing 100021, China; 4 China Anti-Cancer Association Specialty Committee of Glioma, Beijing 100730, China

**Keywords:** ^1^H-MRS, choline, metabolites, glioma, prognostic prediction

## Abstract

Glioma is the most prevalent primary central nervous system malignant tumor, with high heterogeneity observed among different grades; therefore, non-invasive prediction of prognosis could improve the clinical management of patients with glioma. ^1^H-magnetic resonance spectroscopy (MRS) can estimate metabolite levels non-invasively. Multiple studies have investigated its prognostic value in gliomas; however, no consensus has been reached. PubMed and Embase databases were searched up to 20 October 2022 to identify studies investigating the prognostic value of metabolites using ^1^H-MRS in patients with glioma. Heterogeneity across studies was evaluated using the Q and *I*^2^ tests, and a fixed- or random-effects model was used to estimate the combined overall hazard ratio (HR). Funnel plots and Begg tests were used to assess publication bias. Higher choline levels were associated with shorter overall survival (HR = 2.69, 95% CI, 1.92–2.99; *p* < 0.001) and progression-free survival (HR = 2.20, 95% CI, 1.16–4.17; *p* = 0.02) in all patients; however, in pediatric gliomas, it showed no significant correlation with overall survival (HR = 1.60, 95% CI, 0.97–2.64; *p* = 0.06). The estimated choline level by ^1^H-MRS could be used to non-invasively predict the prognosis of patients with adult gliomas, and more studies are needed to evaluate the prognostic value of other metabolites.

## 1. Introduction

Gliomas are the most prevalent primary malignant tumors of the central nervous system (CNS) [1]. Despite the utilization of standard Stupp therapy combined with multiple chemotherapies, targeted therapy, immunotherapy, and tumor-treating field treatment, the overall survival (OS) of patients with glioma remains poor, varying among different World Health Organization (WHO) grades of glioma, with a 5-year survival rate of 7% for glioblastoma, the most aggressive subtype of gliomas [2]. Since there are highly heterogeneous malignancy and survival characteristics among gliomas, the development of a non-invasive tool to predict the prognosis of patients may help with the management of gliomas and improve the survival time and quality of life of patients.

Metabolic alterations within the tumor microenvironment are essential characteristics of cancer because of the high proliferation rate and demand of cancer cells [3]. Multiple studies have shown that metabolic changes in gliomas are associated with tumor grades [4], indicating that metabolites might be potential predictive biomarkers of prognosis. ^1^H-magnetic resonance spectroscopy (MRS) can detect various metabolites non-invasively and can provide estimated levels of choline (Cho), creatine (Cr), N-acetyl aspartate (NAA), lactate (Lac), and lipids [5]. Recent studies have shown that 2-hydroxyglutarate (2-HG), which is a product of isocitrate dehydrogenase (IDH)-mutant glioma, plays a key role in cancer metabolism reprogramming [6], with particular attention to metabolic alterations in the tumor microenvironment of glioma.

Multiple studies have investigated the potential prognostic value of metabolites estimated using ^1^H-MRS, and several potential biomarkers have been identified [3,5,7,8,9,10,11,12,13,14,15,16,17,18], such as Cho/Cr, Cho/NAA, and 2-HG. However, some of the results of these studies were contradictory, and no consensus has been reached. Therefore, in this meta-analysis and systematic review, we aimed to investigate whether various metabolites measured using ^1^H-MRS could predict prognosis non-invasively in patients with glioma.

## 2. Materials and Methods

### 2.1. Literature Search and Selection of Studies

The current meta-analysis was conducted in accordance with the guidelines of the Preferred Reporting Items for Systematic Reviews and Meta-Analyses (PRISMA) [19]. PubMed and EMBASE were used to search for studies exploring the prognostic value of metabolites measured using MRS in gliomas up to 20 October 2022. The following search strategies were used: ((MR spectroscopy) OR (MRS)) AND (glioma) AND (survival) AND ((predict) OR (prognostic risk)). We also searched for potential studies by screening citations of the included studies and reviews. The search was limited to studies published in English. The protocol and systematic search strategy of the review are documented online (CRD42022368691) in the International Prospective Register of Systematic Reviews Registry (PROSPERO).

### 2.2. Eligibility Criteria

The search results were first screened for titles and abstracts, and further evaluated based on a full-text review. Three authors (Y.S., D.L., and Z.K.) independently assessed the search results for study inclusion, discussed potentially controversial studies, and reached an agreement.

Studies were considered eligible for inclusion if all the following criteria were met: (1) patients had preoperative or postoperative in vivo ^1^H-MRS, (2) patients had histopathologically confirmed WHO grade 2–4 glioma, (3) patient outcomes were defined as OS or progression-free survival (PFS), and (4) the prognostic value of metabolites was measured using ^1^H-MRS and evaluated with hazard ratio (HR) and 95% confidence interval (95% CI), or if there was sufficient data to calculate HR and 95% CI.

Studies were excluded if they entailed any of the following criteria: (1) a review article or conference abstract, (2) letters, editorials, and comments, (3) animal or in vitro studies, (4) studies with partially overlapping cohort data, and (5) studies assessing treatment response. For studies with overlapping data, the study with the completed study results was selected.

### 2.3. Data Extraction and Quality Assessment

Three authors (Y.S., D.L., and Z.K.) independently extracted information using a standardized extraction form, including study and patient characteristics, and MRI characteristics of the selected studies. Firstly, the study and patient characteristics were obtained: author, year of publication, country, study design, number of patients, glioma subtypes, WHO grade of glioma, primary or recurrent gliomas, the median age of patients, and male/female ratio of patients. Secondly, MRI characteristics were obtained: magnetic strength, MRS techniques, echo time (TE/ms), software for postprocessing of MRS imaging, the timing of MRS, metabolites utilized to predict prognosis, the cutoff value, and the assessment of outcomes. (The latter included HR and the corresponding 95% CI of PFS and/or OS. If they were not given, particularly in an article, the essential data that were used to estimate them were collected. The estimation methods used were reported by Tierney et al. [20]). If the results of the univariate and multivariate analyses were both stated in a previous study, the multivariate analysis was included in the analysis.

The quality of the enrolled studies was assessed using Newcastle-Ottawa Scale (NOS) for cohort studies [21]. Cohort selection (score 0–4), comparability (score 0–2), and outcome (0–3) were independently evaluated by two authors (Q.L. and H.X.), with a total score of 0–9 for each study. If a disagreement occurred, a third author (Y.W.) assessed the study and reached a consensus.

### 2.4. Statistical Analyses

The heterogeneity of HRs across studies was evaluated using Q and *I*^2^ statistics. If there was no significant heterogeneity across the studies (*I*^2^ < 50%, *p* > 0.1), fixed-effects models were used for the combined risk estimates. If there was significant heterogeneity (*I*^2^ ≥ 50%, *p* ≤ 0.1), random effects models were used. Sensitivity analysis was used to assess the potential reasons for heterogeneity and verify the reliability of the results. Potential publication bias was qualitatively evaluated using a funnel plot and quantitatively evaluated using the Begg test. Review Manager 5.4.1 (The Cochrane Collaboration, London, UK, 2020) and STATA version 17.0 (StataCorp LLC, College Station, TX, USA) were used for statistical analyses. A *p*-value < 0.05 was considered statistically significant.

## 3. Results

### 3.1. Literature Search

**Figure 1:**
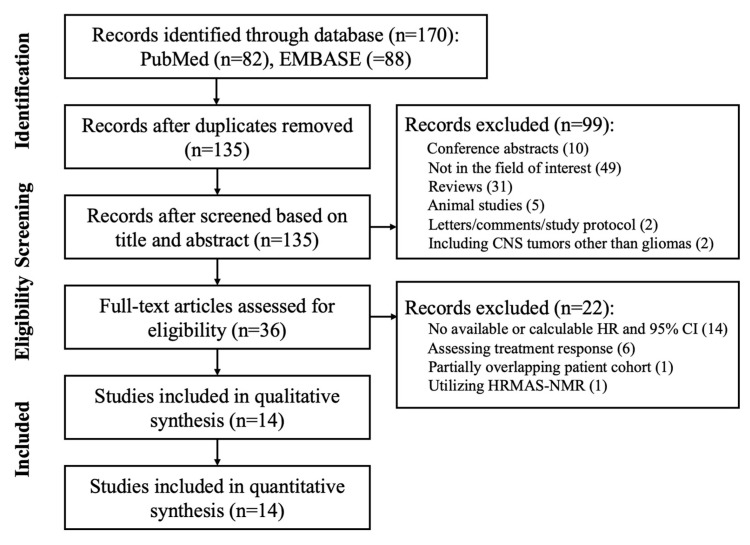
Flow diagram of the study process.

A total of 170 studies were identified through a database search, and after removing duplicates, 135 studies were screened based on titles and abstracts and evaluated based on full-text articles according to our eligibility criteria. A total of 121 studies were excluded for the following reasons: 10 were conference abstracts, 49 were not in the fields of interest, 31 were reviews, 5 were animal studies, 2 were letters/study protocols, 2 included central nervous system (CNS) tumors other than gliomas, 14 had no available or calculable HR and corresponding 95% CI, 6 assessed treatment response, 1 analyzed partially overlapping patient cohorts, and 1 utilized HRMAS-NMR (Figure 1). In total, 14 studies with 568 patients investigating the prognostic value of ^1^H-MRS were included in the final meta-analysis and systemic review.

### 3.2. Study Characteristics

The NOS scores for the 14 high-quality studies ranged from 7–9 (Supplementary Table S1). These studies were published between 2000 and 2022, of which six were conducted in the USA, two in China, one in Canada, one in Germany, one in Japan, one in Mexico, one in Poland, and one in Sweden. A total of 568 patients were included in the current analysis and review, with a median age ranging from 13.5 to 57 years. Some studies focused on pediatric gliomas, particularly diffuse intrinsic pontine glioma (DIPG), and others enrolled patients with adult glioma, including astrocytoma, oligodendroglioma, and glioblastoma, varying from WHO grades 2–4 (Table 1).

The parameter and post-processing software used in the selected studies varied, and the magnetic strength and echo time (TE) chosen were mostly 3T and 144ms, respectively. All studies utilized ^1^H-MRS and point-resolved spectroscopic selection (PRESS) to detect metabolites, including Cho, lactate, Cr, NAA, glycine, and 2-HG, of which nine studies considered the prognostic value of Cho/Cr and/or Cho/NAA. Thirteen studies evaluated the predictive value of metabolites for OS and four studies investigated PFS (Table 2).

### 3.3. Choline and Overall Survival

**Figure 2:**
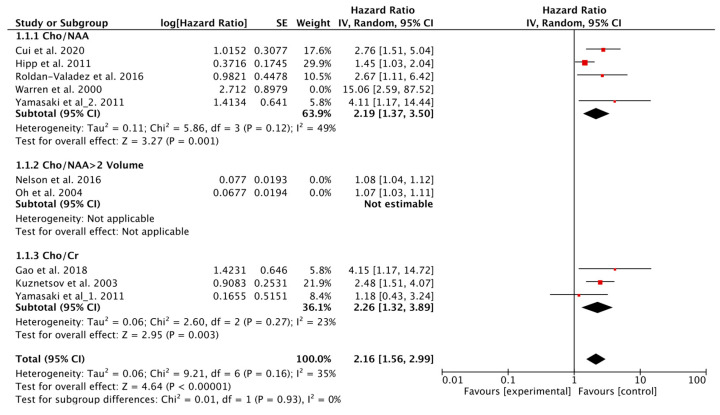
Forest plots of the pooled hazard ratio for OS of choline.

**Figure 3:**
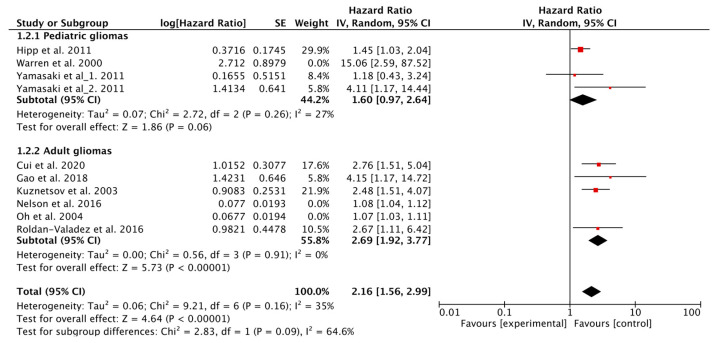
Forest plots of the subgroup analysis of OS in the selected studies using choline as parameters.

In total, nine studies evaluated the value of Cho as a prognostic biomarker of OS in patients with glioma [5,7,9,10,12,13,14,16,17], of which five utilized Cho/NAA as a predictive parameter [7,12,13,14,17], two used the volume of the region with Cho/NAA > 2 [5,10], and three used Cho/Cr [9,13,16]. The Q test and *I*^2^ statistic showed *p* < 0.1, *I*^2^ = 80%; therefore, a random-effects model was used. The results indicated that higher Cho levels were associated with worse OS (HR = 1.30, 95% CI, 1.14–1.49; *p* < 0.001). Subgroup analysis showed that lower Cho/NAA, the volume of regions with Cho/NAA > 2, and Cho/Cr all indicated shorter OS (HR = 2.72, 95% CI, 1.51–4.90; *p* < 0.001; HR = 1.08, 95% CI, 1.05–1.10; *p* < 0.001; HR = 2.26, 95% CI, 1.32–3.89; *p* < 0.001). Heterogeneity was observed across all studies in this analysis (*p* < 0.1, *I*^2^ = 80%), and a sensitivity analysis was conducted to explore potential reasons for heterogeneity. Since the study conducted by Warren et al. focused on pediatric patients with recurrent glioma after various treatments, heterogenity may be induced into the analysis. The exclusion of studies utilizing the volume of the region with Cho/NAA > 2 as a predictor [5,10], and that conducted by Warren et al. [7], yielded better results (HR = 2.16, 95% CI, 1.56–2.99; *p* < 0.001) with no observed heterogeneity (*p* = 0.16, *I*^2^ = 35%) (Figure 2). After exclusion, further subgroup analysis based on glioma subtypes was conducted, and the results showed that in adult gliomas, higher Cho indicated worse OS (HR = 2.69, 95% CI, 1.92–2.99; *p* < 0.001). Whereas, the association between Cho and OS was inconclusive in pediatric gliomas (HR = 1.60, 95% CI, 0.97–2.64; *p* = 0.06), and subgroup differences were identified (*p* < 0.1, *I*^2^ = 64.6%) (Figure 3).

### 3.4. Choline and Progression-Free Survival

**Figure 4:**
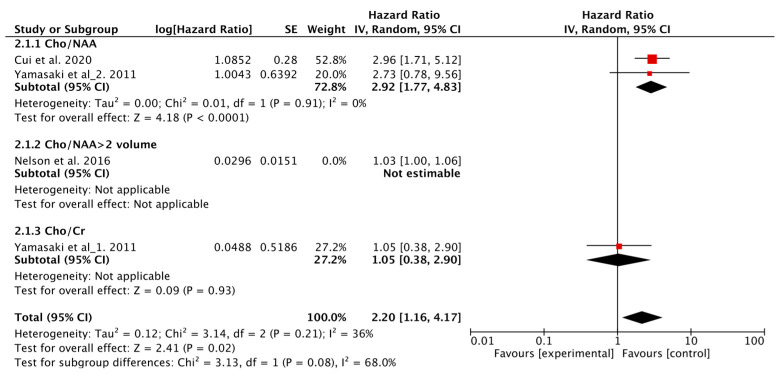
Forest plots of the pooled hazard ratio for PFS of choline.

In total, three studies evaluated the associations between Cho and PFS [5,13,17], two studies used Cho/NAA as the predictive parameter, one study assessed Cho/Cr, and one study assessed the volume of regions with Cho/NAA > 2. The Q test and *I*^2^ statistic showed *p* < 0.1, *I*^2^ = 82%; therefore, a random-effects model was used. The results showed no significant association between Cho and PFS (HR = 1.64, 95% CI, 0.82–3.28; *p* < 0.001). Since heterogeneity was observed, sensitivity analysis was conducted, and when studies using Cho/NAA and Cho/Cr were included, no heterogeneity was observed across studies (*p* = 0.21, *I*^2^ = 36%) and better results were obtained, suggesting that Cho was associated with shorter PFS (HR = 2.20, 95% CI, 1.16–4.17; *p* = 0.02) (Figure 4).

### 3.5. The Prognostic Value of Other Metabolites Measured Using ^1^H-MRS

After excluding studies evaluating the prognostic value of Cho in gliomas, there was not enough data to conduct a meta-analysis of other metabolites, including Cr [11], lipid-lactate [14], lactate [8,9,13], myo-inositol [15], glycine [3], 2-HG [3,18], and glutamate [18]. The associations between these metabolites and prognoses are summarized in Table 3.

### 3.6. Publication Bias

**Figure 5:**
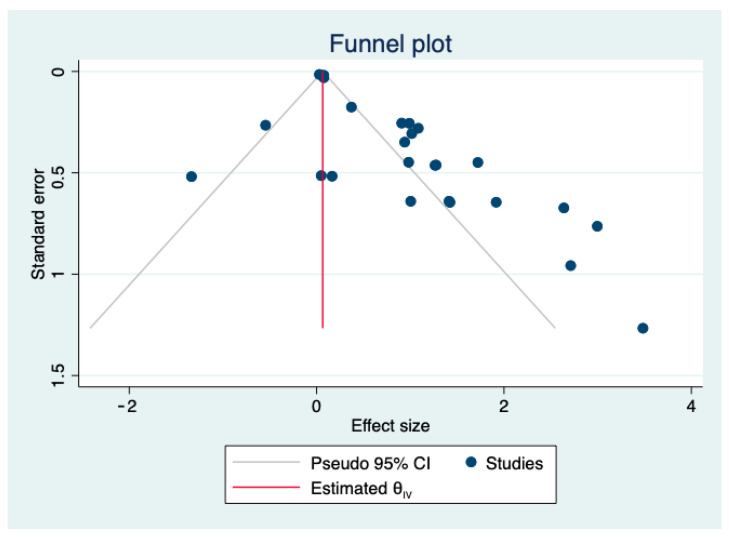
Funnel plot of the selected studies assessing publication bias.

Potential publication bias was assessed using a funnel plot and Begg’s test (Figure 5). The funnel plot showed moderate asymmetry; however, Begg’s test showed no evidence of publication bias among the included studies (*p* = 0.0856).

## 4. Discussion

This study suggests that Cho levels estimated by ^1^H-MRS have great prognostic value in gliomas, both for OS and PFS. Subgroup analysis showed that Cho level is less predictive of survival in pediatric gliomas. Lower Cho/NAA, the volume of regions with Cho/NAA > 2, and Cho/Cr are all associated with shorter OS. Previous studies have confirmed that Cho is associated with membrane synthesis and degradation. NAA represents a neuronal function, and Cr participates in energy metabolism [22,23]. Higher levels of both Cho/NAA and Cho/Cr indicate CNS tumor malignancy and distinguish the WHO grades of gliomas [4]. A recent study conducted by Gao et al. [16] suggested the Cho/Cr ratio as a biomarker for cellular proliferation and that it could be used to predict the prognosis of glioma. Additionally, Pucci et al. [24] showed that Cho could promote the proliferation of glioblastoma cells by activating the AKT and ERK pathways, suggesting that it contributes to the aggressiveness of glioblastoma. Despite the use of MRS as a non-invasive estimate of Cho, quantitative features from CHO PET were also investigated and found to be effective in distinguishing the WHO grades of glioma [25] and predicting molecular alterations in glioma [26].

In pediatric gliomas, particularly DIPG, Hipp et al. [12] and Yamasaki et al. [13] found that Cho/NAA was prognostic. Yet, Cho/Cr showed no significant value in predicting OS and PFS, which might partially explain why our analysis showed no prognostic value for Cho in pediatric gliomas. However, further studies regarding the function of Cho in pediatric gliomas are lacking, and more studies are warranted to determine the use of Cho as a predictive biomarker and explain the underlying molecular mechanism.

In the WHO 2021 guidelines for CNS tumors, molecular alterations have been regarded as an essential part of glioma classification [27], and 2-HG holds considerable importance because it is the oncometabolite of IDH mutation [28]. 2-HG competitively inhibits α-ketoglutarate-dependent dioxygenases, leading to epigenetic disorders and cell differentiation blocks, and can also improve HIF-1 α level, induce angiogenesis, and mediate the escape and metastasis of tumor cells [29]. Consistent with clinical experience, Tiwari et al. [3] found that lower survival risk was associated with higher levels of 2-HG and the presence of IDH mutations. Interestingly, Autry et al. [18] revealed that higher levels of 2-HG/Cr indicated a significant reduction in PFS, which contradicts our clinical impression. However, this study enrolled patients with IDH mutations, suggesting that in patients with IDH mutation, 2-HG levels might play an adverse role in survival, and a more complicated metabolic network might be involved in glioma growth.

Recently, more investigations on metabolic heterogeneity of glioblastoma utilizing MRS have been conducted. Grande et al. applied MRS in glioblastoma stem-like cells and detected multiple metabolites in vitro, indicating an important role of mitochondrial fatty oxidation in energy supplements in glioblastoma stem-like cells [30]. They also discovered specific metabolic signatures of glioblastoma stem-like cells after stressful treatments via MRS detection [31]. All the studies suggest more advanced utilization of MRS in metabolic investigations and clinical practice, and further investigations could be conducted.

Our study has some limitations. Firstly, we included several retrospective studies, and the baseline characteristics of enrolled patients were not steady, hence making it vulnerable to selection bias. The variety of device parameters and selected samplings would result in potential bias and heterogeneity, and well-designed prospective studies may be conducted to further evaluate the prognostic value of MRS. Additionally, since the studies selected in our meta-analysis were between 2000 and 2022, there are improvements in the management of glioma-induced heterogeneity. Although we conducted a subgroup analysis for essential factors, other confounding factors remained. Finally, owing to the limited number of studies, several subgroups could not be included in the meta-analysis, such as the predictive value of Cho for PFS, the prognostic value of other metabolites, and the predictive value of Cho in pediatric low-grade glioma and high-grade glioma. Thus, more studies to investigate the remaining problem are warranted. Therefore, further studies are needed to determine other metabolites measured by ^1^H-MRS as prognostic biomarkers.

## 5. Conclusions

The estimated level of Cho by ^1^H-MRS could be used to non-invasively predict the prognosis of patients with adult gliomas, and its use in predicting the survival time of pediatric patients with glioma should be carefully considered. More studies are needed to evaluate the prognostic value of other metabolites such as 2-HG, glycine, and lactate.

## Figures and Tables

**Table 1:** Study and patient characteristics of the selected studies.

Author (Year of Publication)	Country	Study Design	Number of Patients	Glioma Subtype	WHO Grade	Primary or Recurrent	Median Age (Range)	Male/Female Ratio
Warren et al. (2000)	USA	Prospective	27	Pediatric glioma ^a^	Mix	Recurrent	14 (5–20)	NA
Tarnawski et al. (2002)	Poland	Prospective	51	Adult glioma ^b^	High grade	Primary	47 (20–68)	35:16
Kuznetsov et al. (2003)	Canada	Retrospective	54	Adult glioma	Low grade	Primary	45.7 (19–82)	NA
Oh et al. (2004)	USA	Prospective	28	Adult glioma	High grade	Primary	53.7 (14.6–79.6)	NA
Hattingen et al. (2010)	Germany	Retrospective	61	Adult glioma	Low grade	Primary	38 (20–66)	37:24
Hipp et al. (2011)	USA	Prospective	34	Pediatric glioma	Mix	Primary	5.5 (1.6–14.6)	12:22
Yamasaki et al. (2011)	Japan	Retrospective	19	Pediatric glioma	Mix	Primary	13.5 (4–36)	10:9
Roldan-Valadez et al. (2016)	Mexico	Retrospective	28	Adult glioma	High grade	Primary	50 (13–85)	9:19
Nelson et al. (2016)	USA	Prospective	43	Adult glioma	High grade	Primary	57 (27–80)	NA
Durmo et al. (2018)	Sweden	Retrospective	33	Adult glioma	Mix	Primary	57 (27–77)	11:22
Gao et al. (2018)	China	Retrospective	43	Adult glioma	Mix	Primary	47 (8–66)	28:25
Cui et al. (2020)	China	Retrospective	67	Adult glioma	High grade	Primary	47.1 (25.5–58.7)	41:26
Tiwari et al. (2020)	USA	Prospective	35	Adult glioma	Mix	Primary	39 (21–79)	19:16
Autry et al. (2022)	USA	Prospective	45	Adult glioma	Low grade	Mix	34 (19–72)	33:12

^a^ Pediatric glioma includes diffuse intrinsic pediatric glioma. ^b^ Adult glioma includes astrocytoma, oligodendroglioma, and glioblastoma.

**Table 2:** MRI and metabolite characteristics of the selected studies.

Author (Year of Publication)	Magnet Strength (T)	Vendor	MRS Techniques	TE (ms)	Software	Timing of MRS	Adjusted Factors	Parameter	Cutoff
Warren et al. (2000)	1.5	GE	PRESS ^a^	NA	Sun Workstation	Post-treatment	None	Cho/NAA	4.5
Tarnawski et al. (2002)	2	Elscint	PRESS	35	NA	Pre-surgery	Age	Lac/NAA	2
Kuznetsov et al. (2003)	1.5	Philips	PRESS	272	AVIS, MNI/H	Pre-surgery	Low NA/Cr voxels	Cho/Cr	NA
								Lac/Cr	NA
Oh et al. (2004)	1.5	GE	PRESS	144	NA	Post-surgery	Age	Volume of Cho/NAA > 2	15.7
Hattingen et al. (2010)	3	Siemens	PRESS	30 144	LCModel	Pre-surgery	None	Cr	0.93
Hipp et al. (2011)	1.5	GE	PRESS	280	GE Software	Post-surgery	None	Cho/NAA	NA
Yamasaki et al. (2011)	3	GE	PRESS	30	GE Software	Pre-surgery	None	Cho/Cr	2
								Cho/NAA	2
								Lactate	Present
Roldan-Valadez et al. (2016)	3	GE	PRESS	26 144	Func Tool	Pre-surgery	Age	Cho/NAA	NA
								LL/Cr ^b^	NA
Nelson et al. (2016)	3	GE	PRESS	144	Linux Workstation	Post-surgery	None	Volume of Cho/NAA > 2	NA
Durmo et al. (2018)	3	Siemens	PRESS	144	LCModel	Pre-surgery	None	Ins/Cho	1.29
Gao et al. (2018)	3	Siemens	PRESS	135	Siemens Platform	Pre-surgery	MCM2 labeling index	Cho/Cr	2.68
Cui et al. (2020)	3	Siemens	PRESS	135	NA	Post-surgery	Radiotherapy, MGMT methylation	Cho/NAA	1.31
Tiwari et al. (2020)	3	Philips	PRESS	97	Philips Platform	Pre-surgery	None	2-HG	1
								Glycine	2.5
								Glycine/2-HG	2.5
Autry et al. (2022)	3	GE	PRESS	32 65	LCModel	Pre-surgery	Tumor volume, tumor enhancement	2-HG/Cr	0.905
								Glu/Cr	0.945

^a^ Point-resolved spectroscopic selection. ^b^ The ratio of lactate and lipid to choline.

**Table 3:** Other metabolite parameters in the selected studies.

Author (Year of Publication)	Parameter	Cutoff	Overall Survival		Progression Free Survival	
			Hazard Ratio (HR)	95% CI ^a^	Hazard Ratio (HR)	95% CI ^a^
Tarnawski et al. (2002)	Lac/NAA	2	14.00	3.74–52.35	NA	NA
Kuznetsov et al. (2003)	Lac/Cr	NA	2.69	1.63–4.44	NA	NA
Hattingen et al. (2010)	Cr	0.93	1.08	1.02–1.15	NA	NA
Yamasaki et al. (2011)	Lactate	Present	3.54	1.43–8.78	3.58	1.45–8.86
Roldan-Valadez et al. (2016)	LL/Cr	NA	0.58	0.35–0.99	NA	NA
Durmo et al. (2018)	Ins/Cho	1.29	2.56	1.29–5.06	NA	NA
Tiwari et al. (2020)	2-HG	1	0.26	0.095–0.73	NA	NA
	Glycine	2.5	6.8	1.92–24.07	NA	NA
	Glycine/2-HG	2.5	20.00	4.48–89.39		
Autry et al. (2022)	2-HG/Cr	0.905	NA	NA	5.59	2.08–12.09
	Glu/Cr	0.945	NA	NA	32.57	2.72–389.94

^a^ 95% confidence interval.

## Supplementary Materials

The following supporting information can be downloaded at: https://www.mdpi.com/article/10.3390/metabo12121219/s1. Table S1: the quality assessment of the selected studies.

## References

[1] Ostrom Q.T., Price M., Neff C., Cioffi G., Waite K.A., Kruchko C., Barnholtz-Sloan J.S. (2022). CBTRUS statistical report: Primary brain and other central nervous system tumors diagnosed in the United States in 2015–2019. Neuro. Oncol..

[2] Miller K.D., Ostrom Q.T., Kruchko C., Patil N., Tihan T., Cioffi G., Fuchs H.E., Waite K.A., Jemal A., Siegel R.L. (2021). Brain and other central nervous system tumor statistics, 2021. CA Cancer J. Clin..

[3] Tiwari V., Daoud E.V., Hatanpaa K.J., Gao A., Zhang S., An Z., Ganji S.K., Raisanen J.M., Lewis C.M., Askari P. (2020). Glycine by MR spectroscopy is an imaging biomarker of glioma aggressiveness. Neuro. Oncol..

[4] Wang Q., Zhang H., Zhang J., Wu C., Zhu W., Li F., Chen X., Xu B. (2016). The diagnostic performance of magnetic resonance spectroscopy in differentiating high-from low-grade gliomas: A systematic review and meta-analysis. Eur. Radiol..

[5] Nelson S.J., Kadambi A.K., Park I., Li Y., Crane J., Olson M., Molinaro A., Roy R., Butowski N., Cha S. (2017). Association of early changes in 1H MRSI parameters with survival for patients with newly diagnosed glioblastoma receiving a multimodality treatment regimen. Neuro. Oncol..

[6] Ježek P. (2020). 2-hydroxyglutarate in cancer cells. Antioxid. Redox Signal..

[7] Warren K.E., Frank J.A., Black J.L., Hill R.S., Duyn J.H., Aikin A.A., Lewis B.K., Adamson P.C., Balis F.M. (2000). Proton magnetic resonance spectroscopic imaging in children with recurrent primary brain tumors. J. Clin. Oncol..

[8] Tarnawski R., Sokol M., Pieniazek P., Maciejewski B., Walecki J., Miszczyk L., Krupska T. (2002). 1H-MRS in vivo predicts the early treatment outcome of postoperative radiotherapy for malignant gliomas. Int. J. Radiat. Oncol. Biol. Phys..

[9] Kuznetsov Y.E., Caramanos Z., Antel S.B., Preul M.C., Leblanc R., Villemure J.G., Pokrupa R., Olivier A., Sadikot A., Arnold D.L. (2003). Proton magnetic resonance spectroscopic imaging can predict length of survival in patients with supratentorial gliomas. Neurosurgery.

[10] Oh J., Henry R.G., Pirzkall A., Lu Y., Li X., Catalaa I., Chang S., Dillon W.P., Nelson S.J. (2004). Survival analysis in patients with glioblastoma multiforme: Predictive value of choline-to-N-acetylaspartate index, apparent diffusion coefficient, and relative cerebral blood volume. J. Magn. Reson. Imaging.

[11] Hattingen E., Delic O., Franz K., Pilatus U., Raab P., Lanfermann H., Gerlach R. (2010). (1)H MRSI and progression-free survival in patients with WHO grades II and III gliomas. Neurol. Res..

[12] Hipp S.J., Steffen-Smith E., Hammoud D., Shih J.H., Bent R., Warren K.E. (2011). Predicting outcome of children with diffuse intrinsic pontine gliomas using multiparametric imaging. Neuro-Oncology.

[13] Yamasaki F., Kurisu K., Kajiwara Y., Watanabe Y., Takayasu T., Akiyama Y., Saito T., Hanaya R., Sugiyama K. (2011). Magnetic resonance spectroscopic detection of lactate is predictive of a poor prognosis in patients with diffuse intrinsic pontine glioma. Neuro-Oncology.

[14] Roldan-Valadez E., Rios C., Motola-Kuba D., Matus-Santos J., Villa A.R., Moreno-Jimenez S. (2016). Choline-to-N-acetyl aspartate and lipids-lactate-to-creatine ratios together with age assemble a significant Cox’s proportional-hazards regression model for prediction of survival in high-grade gliomas. Br. J. Radiol..

[15] Durmo F., Rydelius A., Cuellar Baena S., Askaner K., Lätt J., Bengzon J., Englund E., Chenevert T.L., Björkman-Burtscher I.M., Sundgren P.C. (2018). Multivoxel 1H-MR spectroscopy biometrics for Preoprerative differentiation between brain tumors. Tomography.

[16] Gao W., Wang X., Li F., Shi W., Li H., Zeng Q. (2019). Cho/Cr ratio at MR spectroscopy as a biomarker for cellular proliferation activity and prognosis in glioma: Correlation with the expression of minichromosome maintenance protein 2. Acta Radiol..

[17] Cui Y., Zeng W., Jiang H., Ren X., Lin S., Fan Y., Liu Y., Zhao J. (2020). Higher Cho/NAA ratio in postoperative peritumoral edema zone is associated with earlier recurrence of glioblastoma. Front Neurol..

[18] Autry A.W., Lafontaine M., Jalbert L., Phillips E., Phillips J.J., Villanueva-Meyer J., Berger M.S., Chang S.M., Li Y. (2022). Spectroscopic imaging of D-2-hydroxyglutarate and other metabolites in pre-surgical patients with IDH-mutant lower-grade gliomas. J. Neurooncol..

[19] Liberati A., Altman D.G., Tetzlaff J., Mulrow C., Gøtzsche P.C., Ioannidis J.P., Clarke M., Devereaux P.J., Kleijnen J., Moher D. (2009). The PRISMA statement for reporting systematic reviews and meta-analyses of studies that evaluate health care interventions: Explanation and elaboration. J. Clin. Epidemiol..

[20] Tierney J.F., Stewart L.A., Ghersi D., Burdett S., Sydes M.R. (2007). Practical methods for incorporating summary time-to-event data into meta-analysis. Trials.

[21] Stang A. (2010). Critical evaluation of the Newcastle-Ottawa scale for the assessment of the quality of nonrandomized studies in meta-analyses. Eur. J. Epidemiol..

[22] Chaumeil M.M., Lupo J.M., Ronen S.M. (2015). Magnetic resonance (MR) metabolic imaging in glioma. Brain Pathol..

[23] Peeling J., Sutherland G. (1992). High-resolution 1H NMR spectroscopy studies of extracts of human cerebral neoplasms. Magn. Reson. Med..

[24] Pucci S., Fasoli F., Moretti M., Benfante R., Di Lascio S., Viani P., Daga A., Gordon T.J., McIntosh M., Zoli M. (2021). Choline and nicotine increase glioblastoma cell proliferation by binding and activating α7- and α9- containing nicotinic receptors. Pharmacol. Res..

[25] Kong Z., Jiang C., Liu D., Chen W., Ma W., Cheng X., Wang Y. (2021). Quantitative features from CHO PET distinguish the WHO grades of primary diffuse glioma. Clin. Nucl. Med..

[26] Kong Z., Zhang Y., Liu D., Liu P., Shi Y., Wang Y., Zhao D., Cheng X., Wang Y., Ma W. (2021). Role of traditional CHO PET parameters in distinguishing IDH, tert and MGMT alterations in primary diffuse gliomas. Ann. Nucl. Med..

[27] Louis D.N., Perry A., Wesseling P., Brat D.J., Cree I.A., Figarella-Branger D., Hawkins C., Ng H.K., Pfister S.M., Reifenberger G. (2021). The 2021 WHO Classification of Tumors of the central nervous system: A summary. Neuro. Oncol..

[28] Dang L., White D.W., Gross S., Bennett B.D., Bittinger M.A., Driggers E.M., Fantin V.R., Jang H.G., Jin S., Keenan M.C. (2009). Cancer-associated IDH1 mutations produce 2-hydroxyglutarate. Nature.

[29] Huang L.E. (2019). Friend or foe-IDH1 mutations in glioma 10 years on. Carcinogenesis.

[30] Grande S., Palma A., Ricci-Vitiani L., Luciani A.M., Buccarelli M., Biffoni M., Molinari A., Calcabrini A., D’Amore E., Guidoni L. (2018). Metabolic Heterogeneity Evidenced by MRS among Patient-Derived Glioblastoma Multiforme Stem-Like Cells Accounts for Cell Clustering and Different Responses to Drugs. Stem. Cells Int..

[31] Grande S., Palma A., Luciani A.M., Anello P., Ricci-Vitiani L., Buccarelli M., D’Alessandris Q.G., Pallini R., Guidoni L., Viti V. (2022). Glioblastoma Stem-Like Cells (GSCs) with Mesenchymal Signature: Lipid Profiles of Mobile Lipids Obtained with MRS before and after Radio/Chemical Treatments. Biomolecules.

