# Supplementary material for: Prognostic Value of Choline and Other Metabolites Measured Using 1H-Magnetic Resonance Spectroscopy in Gliomas: A Meta-Analysis and Systemic Review
Source: Metabolites. 2022 Dec 5;12(12):1219. doi: 10.3390/metabo12121219 (PMC9788620; doi:10.3390/metabo12121219)
Supplement: Supplementary file 1 [file metabolites-12-01219-s001.zip › metabolites-2037703-supplementary/Supplementary Table S1_QualityAnalysis.pdf]

Supplementary Table S1 Quality analysis of selected studies utilizing Newcastle-Ottawa Scale (NOS) for cohort studies

	Warren et al. (2000)	Tarnawski et al. (2002)	Kuznetsov et al. (2003)	Oh et al. (2004)	Hattingen et al. (2010)	Hipp et al. (2011)	Yamasaki et al. (2011)	Roldan-Valadez et al. (2016)	Nelson et al. (2016)	Durmo et al. (2018)	Gao et al. (2018)	Autry et al. (2022)	Cui et al. (2020)	Tiwari et al. (2020)
**Selection (0-4)**														
**1. Representativeness of the exposed cohort**														
Truly representative	★					★	★	★	★		★	★		★
Somewhat representative		★	★	★	★					★			★	
Selected group of users, eg nurses, volunteers														
No description of the derivation of the cohort														
**2. Selection of the non-exposed cohort**														
Drawn from the same community as the exposed cohort	★	★	★	★	★	★	★	★	★	★	★	★	★	★
Drawn from a different source														
No description of the derivation of the non exposed cohort														
**3. Ascertainment of exposure**														
Secure record (eg surgical records)														
Structured interview	★	★	★	★	★	★	★	★	★	★	★	★	★	★
Written self report														
No description														
**4. Demonstration that outcome of interest was not present at start of study**														
Yes	★	★		★		★			★			★		★
No			★		★		★	★		★	★		★	
**Comparability (0-2)**														
**1. Comparability of cohorts on the basis of the design or analysis?**														

Study controls for the most important factors	★	★	★	★	★	★	★	★	★	★	★	★	★	★
Study controls for any additional factors		★	★	★	★	★		★			★	★	★	
**Outcome (0-3)**														
**1. Assessment of outcome**														
Independent blind assessment	★			★		★								
Record linkage		★	★		★		★	★	★	★	★	★	★	★
Self report														
No description														
**2. Was follow-up long enough for outcomes to occur?**														
Yes	★	★	★	★	★	★	★	★	★	★	★	★	★	★
No														
**3. Adequacy of follow up of cohorts**														
Complete follow up: all subjects accounted														
Subjects lost to follow up unlikely to introduce bias: > 80% follow up, or description provided of those lost	★	★	★		★	★	★	★	★	★	★	★	★	★
Follow up rate < 80% and no description of those lost				★										
No statement														
**Total Score**	8	9	8	8	8	9	7	8	8	7	8	9	8	8
